# Using K-Nearest Neighbor Classification to Diagnose Abnormal Lung Sounds

**DOI:** 10.3390/s150613132

**Published:** 2015-06-04

**Authors:** Chin-Hsing Chen, Wen-Tzeng Huang, Tan-Hsu Tan, Cheng-Chun Chang, Yuan-Jen Chang

**Affiliations:** 1 Department of Management Information Systems, Central Taiwan University of Science and Technology, Taichung 40601, Taiwan, China; E-Mail: chchen@ctust.edu.tw; 2 Department of Computer Science and Information Engineering, Minghsin University of Science and Technology, Hsinchu 30401, Taiwan, China; E-Mail: wthuang@must.edu.tw; 3 Department of Electrical Engineering, National Taipei University of Technology, Taipei 10608, Taiwan, China; E-Mails: thtan@ntut.edu.tw (T.-H.T.); ccchang@ntut.edu.tw (C.-C.C.); 4 Institute of Biomedical Engineering and Material Science, Central Taiwan University of Science and Technology, Taichung 40601, Taiwan, China

**Keywords:** K-means algorithm, K-nearest neighbor, lung sound, MFCC, stethoscope

## Abstract

A reported 30% of people worldwide have abnormal lung sounds, including crackles, rhonchi, and wheezes. To date, the traditional stethoscope remains the most popular tool used by physicians to diagnose such abnormal lung sounds, however, many problems arise with the use of a stethoscope, including the effects of environmental noise, the inability to record and store lung sounds for follow-up or tracking, and the physician’s subjective diagnostic experience. This study has developed a digital stethoscope to help physicians overcome these problems when diagnosing abnormal lung sounds. In this digital system, mel-frequency cepstral coefficients (MFCCs) were used to extract the features of lung sounds, and then the K-means algorithm was used for feature clustering, to reduce the amount of data for computation. Finally, the K-nearest neighbor method was used to classify the lung sounds. The proposed system can also be used for home care: if the percentage of abnormal lung sound frames is > 30% of the whole test signal, the system can automatically warn the user to visit a physician for diagnosis. We also used bend sensors together with an amplification circuit, Bluetooth, and a microcontroller to implement a respiration detector. The respiratory signal extracted by the bend sensors can be transmitted to the computer via Bluetooth to calculate the respiratory cycle, for real-time assessment. If an abnormal status is detected, the device will warn the user automatically. Experimental results indicated that the error in respiratory cycles between measured and actual values was only 6.8%, illustrating the potential of our detector for home care applications.

## 1. Introduction

Lung auscultation is a diagnostic method used for checking the integrity of lung function. It is a standard preliminary examination for all patients at hospitals, whereby trained physicians use stethoscopes to listen for changes in lung sounds to assess whether a patient has any obvious lung abnormalities. Despite many advances in medical equipment, the traditional analog stethoscope remains the main diagnostic tool used by physicians in lung auscultation.

In modern society, factors such as air pollution, unbalanced diets, excessive stress, and abnormal sleep patterns have resulted in more people suffering from respiratory system diseases. According to recent Department of Health statistics, lung- and respiratory-related diseases ranked fourth and seventh among the top 10 leading causes of death. On average, one person dies from one of these two diseases every two hours [1].

**Figure 1:**
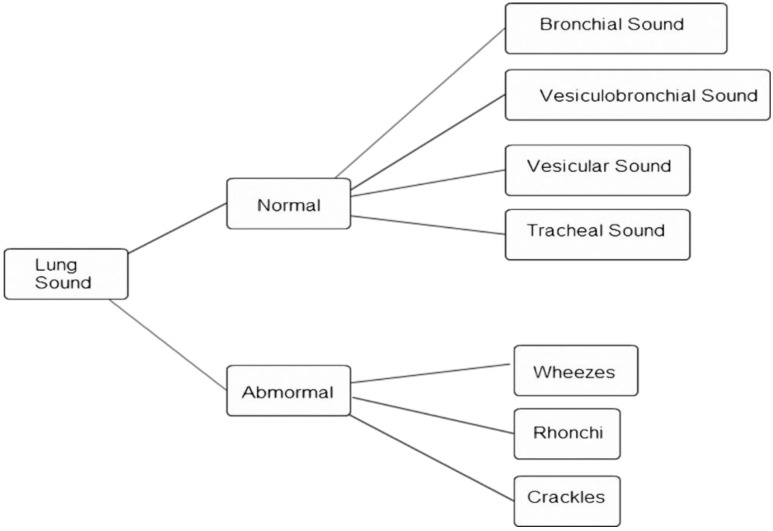
Lung sound classification [2].

Lung sounds can be divided roughly into normal and abnormal sounds, as shown in Figure 1 [2]. Normal breath sounds can be divided into bronchial, vesicular-bronchial, vesicular, and tracheal sounds, while abnormal breath sounds can be divided into crackles, rhonchi, and wheezes. Patients with lung disease have abnormal breath sounds, so abnormal breath sounds are an important component in the diagnosis of lung diseases. Different lung diseases cause different lung sounds: Table 1 lists some associations between abnormal lung sounds and lung diseases [3]. Pneumonia, chronic bronchitis, bronchiectasis, congestive heart failure, and obstructive pulmonary disease produce crackles. Obstructive pulmonary disease, asthma, and bronchial stenosis produce wheezes. Pneumonia, chronic bronchitis, and congestive heart failure produce rhonchi. Researchers have found that the combined population of patients suffering from pneumonia, chronic bronchitis, bronchiectasis, congestive heart failure, obstructive pulmonary asthma, asthma, and bronchial stenosis accounts for about 30% of the global population [4,5,6].

**Table 1:** Associations between abnormal lung sounds and lung diseases [2,3].

Relevant Disease/Abnormal Lung Sound	Crackles	Wheezes	Rhonchi
Pneumonia	●		●
Chronic bronchitis	●		●
Bronchiectasis	●		
Congestive heart failure	●		●
Obstructive pulmonary disease		●	
Asthma		●	
Bronchial stenosis		●	

Many methods for analyzing lung sounds have been proposed. One involves converting lung sounds into a spectrogram: a wheeze-containing lung sound signal will appear as a continuous period of dark blocks in a spectrogram. Thus, an image processing method has been proposed to extract dark blocks to determine the existence of wheezes [7]. Another method involves using mel-frequency cepstral coefficients (MFCCs) to establish normal lung sound and wheeze signal feature models, and to apply vector quantification in signal analysis to determine whether the signals include wheeze signals [8]. A wavelet transformation of lung sound signals has also been proposed, in which eigenvalues are determined to analyze normal lung sound and wheeze signals through a Gaussian mixture model [9]. Another method involves measuring lung sounds by an instrument and collecting breathing flow signals and then applying autoregressive model statistics combined with a nearest neighbor classification (Kth nearest neighbor) to analyze whether the lung sounds are abnormal [10]. One method involves using MFCCs to establish abnormal lung sounds using acoustic feature models, using a Gaussian mixture model to determine whether the sounds are abnormal [11]. With regard to instruments for collecting lung sounds, Suzuki proposed using two groups of condenser microphones to help eliminate background noise: one group of condenser microphones was used to record lung sounds and the other was used to record background noise; an adaptive filter was then used to eliminate background noise from lung sounds [12].

Lung sounds have been widely studied. One report proposed using a PC as a tool for acquisition and analysis, and establishing a user interface for physicians as a diagnostic tool. This hardware used a microphone circuit to sense lung sounds, and a respiratory phase detection circuit detected the breathing state using a thermistor to sense changes in nasal air temperature. The signals were captured by a PC sound card and recorded in the computer [13]. With regard to signal analysis, an electronic stethoscope has been proposed for the auscultation of heart sounds and lung sounds [14]. It uses a condenser microphone with an amplifier, band-pass filter circuit, and shift circuit, and a microprocessor to perform the analog-to-digital conversion. Data are transmitted via a RS232 port to a computer and the sound is played back through speakers. The software LabVIEW was used to develop a user interface to display the lung sound diagrams to provide a diagnosis reference for junior physicians. Another study proposed using computer software to establish wavelet transformations for eigenvalue computation and to identify abnormal breath sounds through an artificial neural network (ANN) [15]. It established a system that could automatically judge relevant symptoms, and its user interface could display the current state of the lung to determine the abnormal lung sounds and symptoms. Another report proposed a digital electronic stethoscope, based on the commercially available digital Walkman [16]. This is a Walkman with a stethoscope head containing an embedded condenser microphone and amplifying and filtering circuits to store a digital lung sound signal. In this system, methods of analysis including fast Fourier transform (FFT) and power spectral density were used to detect wheeze signals. Another study proposed using mel-frequency cepstral coefficients (MFCCs) to capture lung sound characteristics, and then using dynamic time warping to divide the lung sounds into normal sounds, wheezes, and crackles [17]. By enhancing lung sound signals, a dual-sensor spectral subtraction algorithm was used to reduce interfering noise in lung sound identification when capturing the characteristics of lung sound signals in this system. Another study proposed using hidden Markov models to establish feature models of the collected lung sounds of emphysema patients and normal lung sounds [18]. The differences were used to detect whether the abnormal threshold was reached. Other researchers have proposed using neural network (NN) classification technology for lung sound analysis [19]. 

## 2. Materials and Methods

### 2.1. System Design 

**Figure 2:**
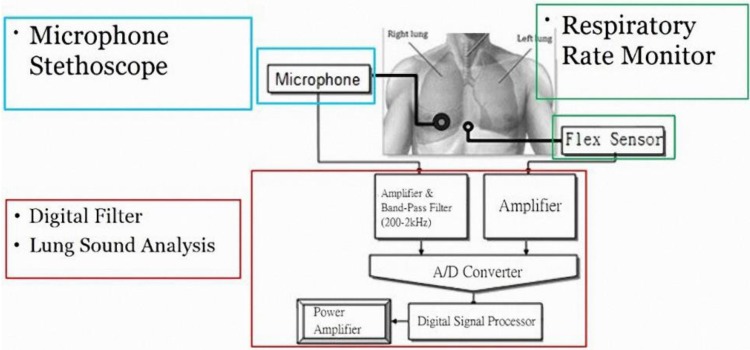
System architecture.

Figure 2 presents the proposed system architecture, which consists of a condenser microphone, digital filter, lung sound analysis system, and respiratory rate monitor. The functions of each unit are described below.

#### 2.1.1. Condenser Microphone

Records lung sound signals measured by the analog stethoscope, amplifies the signals, and transmits them to the computer.

#### 2.1.2. Digital Filter

First, line filtering is conducted with a 10-order Chebyshev II IIR filter; the band-pass range is designed to cover the major frequency range of lung sounds, 200–2000 Hz. Then, wavelet technology is applied to de-noise the signals. The de-noising process can be divided into three major steps:

(a) Select an appropriate wavelet function and determine the number of wavelet decomposition layers M, then conduct the M-layer wavelet decomposition of the original one-dimensional signal S.
(b) For the high-frequency coefficient of each layer of 1-M (j = 1, 2, ..., M), select a threshold T for quantitative processing to get useful high-frequency components. We adopt a soft threshold for quantification by comparing the absolute value with the threshold. Points below or equal to threshold became 0, and points greater than threshold become the difference between the point and the threshold. The mathematical equation is shown in Equation (1) [20]:
  (1) $${\overset{\wedge }{w}}_{j}(t)=\left\{ \begin{array}{ll} \mathrm{sgn}(w_{j}(t))(\left|w_{j}(t)\right|-T) & \text{, }|w_{j}(t)|>T\\ 0 & \text{, }\left|w_{j}(t)\right|\leq T \end{array}\right.$$
(c) Based on the M-layer low-frequency coefficient of the wavelet decomposition and the high-frequency coefficient from the first layer to the M layer after the quantification processing, we can reconstruct the signals to obtain de-noised signals.

**Figure 3:**
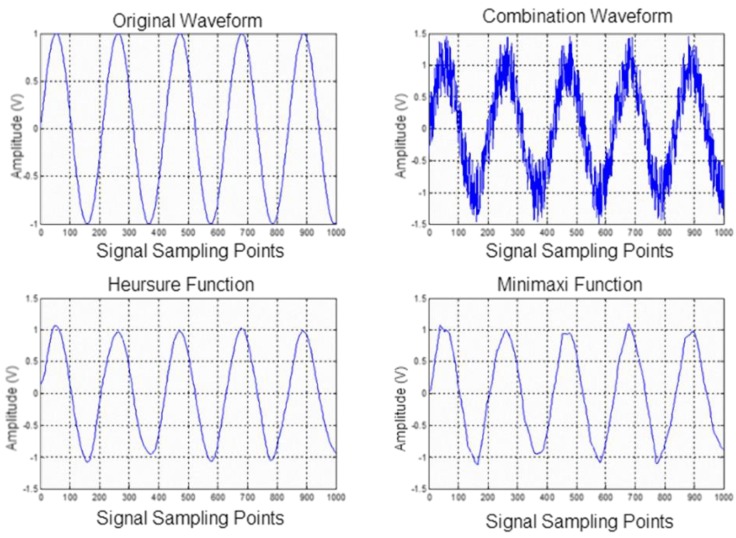
Processing effects of wavelet de-noising for the same case with different threshold values (*M* = 6).

For our analysis, we followed the above steps and used a sine wave to simulate the lung sound signal S. Using the heuristric threshold selection heursure method and the maximum and minimum threshold selection ‘minimaxi’ method, we conducted a wavelet decomposition of signal S. We found that the signal reconstructed waveform was the most complete when the number of wavelet decomposition layers was 6 (*M* = 6) without distortion. Figure 3 presents the results; it shows that the two processing methods can both be used to essentially eliminate the noise interference. However, the minimaxi method may also eliminate some useful signals, resulting in distortion of the signals. This problem did not arise in the heursure method, so we used the heuristric threshold method to select threshold values.

#### 2.1.3. Lung Sound Signal Analysis

To detect abnormal lung sounds, the system uses MFCCs to capture normal and abnormal lung sound (crackle, wheezes, and rhonchi) signal characteristic parameters. Coupled with the K-means algorithm, the signal characteristic parameters are clustered to reduce the amount of data and computation time. Finally, abnormal lung sound signals are classified using a Kth nearest neighbor classification.

#### 2.1.4. Respiratory Rate Monitor

**Figure 4:**
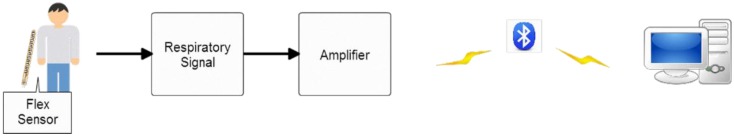
Process of respiratory signal sensing.

The system uses bending sensors to compute the respiratory rate, by measuring belly bulge and bend times. When sensors are placed on the abdomen, the belly bulge resistance of inspiration differs from that of expiration, so this feature is used to monitor the respiratory rate (Figure 4). Sensor signals are amplified appropriately and used as inputs for signal processing, and the processed signals are then transmitted via Bluetooth to a computer to calculate the number of breaths.

#### 2.1.5. Graphical User Interface (GUI) Design of the Diagnosis System

**Figure 5:**
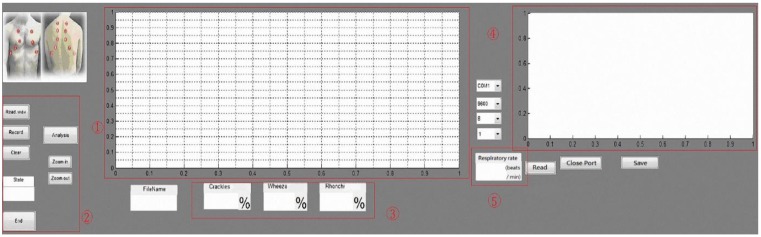
GUI design of the diagnosis system.

Figure 5 shows the GUI for the lung sound classification and identification diagnosis system. In the diagnosis system GUI, Area 1 displays the lung sound waveform, Area 2 is the lung sound file control button, Area 3 contains lung sound abnormal classification results, Area 4 is the respiratory rate waveform, as measured by the bending sensors, and Area 5 is the respiratory cycle (times/min).

### 2.2. Implementation of the Integrated Stethoscope and Respiratory Rate Sensor System 

**Figure 6:**
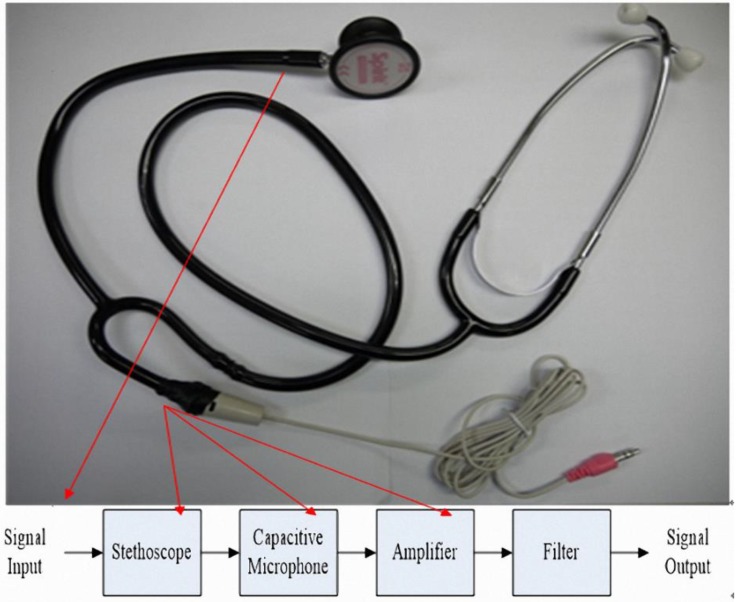
Modified microphone stethoscope.

**Figure 7:**
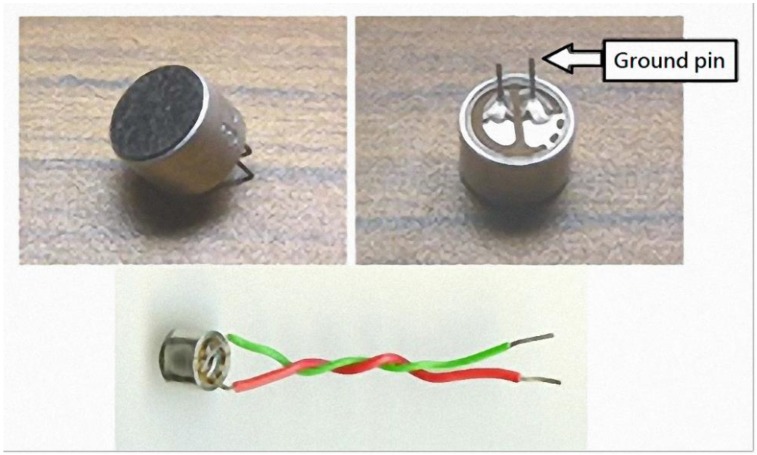
Condenser microphone [21].

The system integrates a traditional analog stethoscope and a condenser microphone to measure and record patient lung sound signals. To do this, we cut out a section of the Y-shaped hose and integrated the condenser microphone and stethoscope head. The microphone circuit was connected to the end of the hose by hot plastic, and wrapped with isolation sticky paper to fill the cracks (Figure 6). Figure 7 shows the implementation of the condenser microphone. The analog stethoscope measures lung sound signals, the stethoscope head collects the sounds, and the microphone records the sounds, which are transmitted to a PC for storage and playback.

### 2.3. Respiratory Rate Sensor 

**Figure 8:**
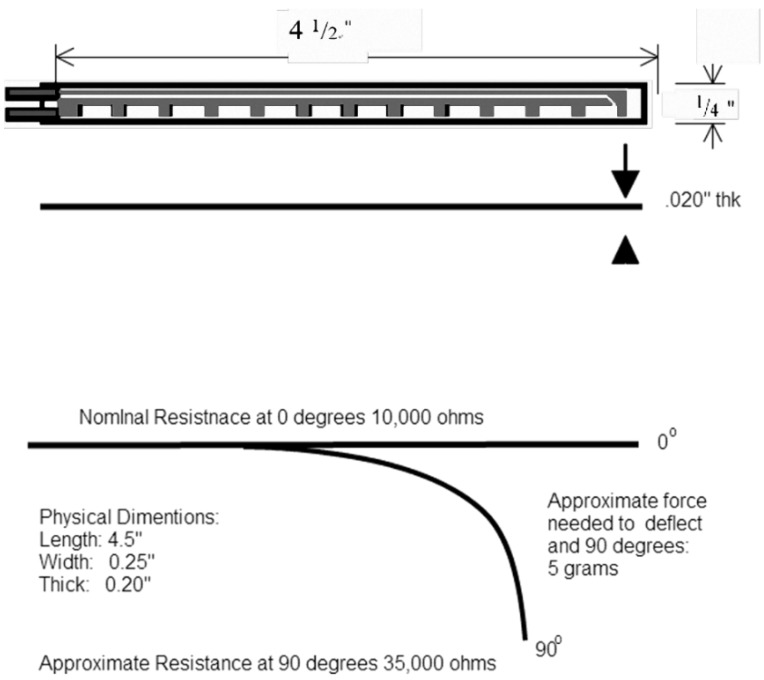
Characteristics of bending-type sensor resistance [22].

The bending-type sensor used was the Flex Sensor (American Images Company [21]). The bending-type sensor is a long, thin sheet of a variable resistor (4.5" long, 0.25" wide, and 0.2" high; see Figure 8). When the bending-type sensor bends, resistance differs according to the degree of bending; resistance ranges from 10–40 kΩ.

**Figure 9:**
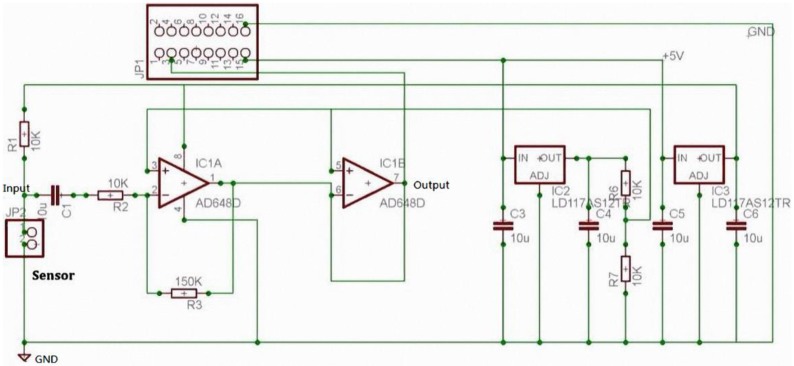
Respiratory sensor circuit.

**Figure 10:**
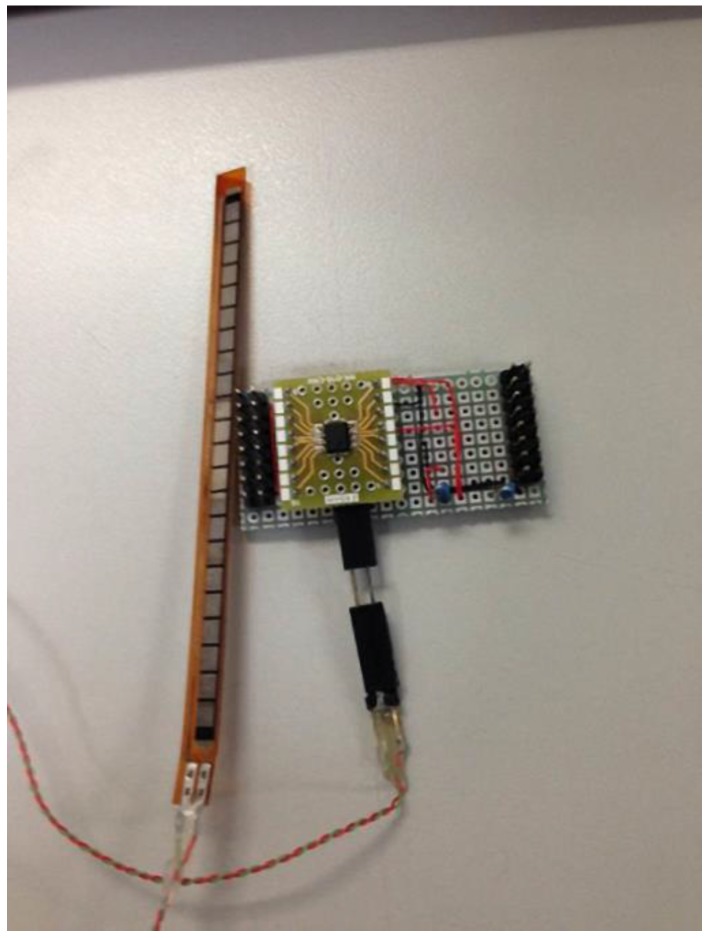
Implementation of respiratory sensor.

Figure 9 illustrates the respiratory rate sensor circuit, which uses an amplifier to amplify the sensing signals. The circuit output equation is shown in Equation (2), and respiratory sensor implementation is shown in Figure 10.

(2) $$V_{out}=-\frac{R_{f}}{R_{in}}V_{in}$$

### 2.4. Implementation of Lung Sound Classification System 

**Figure 11:**
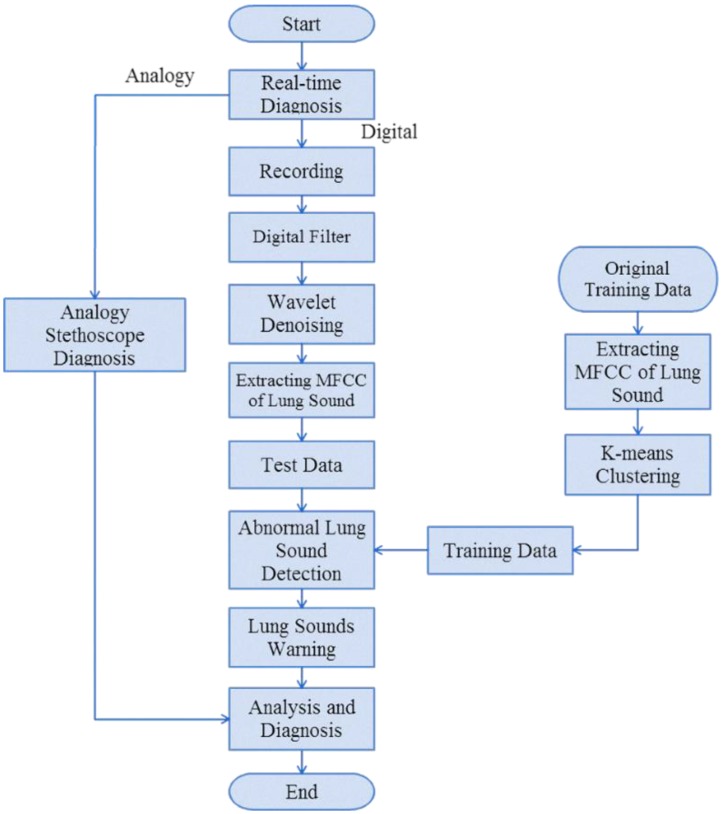
Process of lung sound identification system.

Figure 11 shows the process of the lung sound classification identification system. Mel-frequency cepstral coefficients (MFCCs) capture normal and abnormal lung sound (crackle, wheeze, and rhonchi) characteristic parameters. Coupled with the K-means algorithm, clustering of characteristic parameters reduce the data and computation requirements. Finally, abnormal lung sound signals are classified using a nearest neighbor classification (Kth nearest neighbor). The system theory is described below.

### 2.5. Lung Sound Signal Characteristics Extraction 

**Figure 12:**
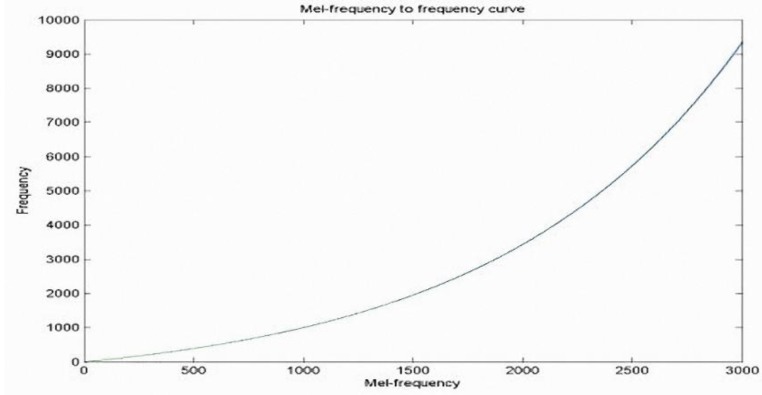
Relationship between human perception and actual frequency.

**Figure 13:**
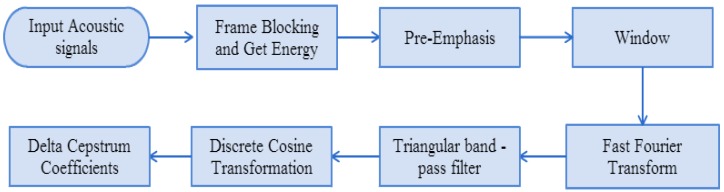
Process of MFCC characteristic parameter capture.

MFCC is now being widely used in speech research and speaker identification systems [9]. It has strong low-frequency sound capabilities, and weaker high-frequency sound perception. Figure 12 illustrates the relationship between human perception of frequency and actual frequency. The MFCC characteristics capture method is based on a Fourier transform: eigenvectors are extracted from the frequencies of each sound frame of the sound signals. Figure 13 presents the MFCC parameter-capturing process, which is described below.

(1) Frame blocking: To observe sound signal characteristics, we collect a certain number of sampling points of signals for observation, referred to as framing. To limit signal changes between frames, frames are generally overlapped in the proportion of 1/2.
(2) Compute energy: After framing, the energy of each frame is computed as the 13th parameter of MFCC:
  (3) $$energy=\sum_{n=1}^{framesize}S{\left(n\right)}^{2}$$
(3) Pre-emphasis: After the sound is sent, the higher-frequency part of the sound will be attenuated. Thus, the pre-emphasis method is used to compensate for the attenuated high-frequency part in identification or comparison. Pre-emphasis compensates for the loss of high-frequency by passing the sound signal through a high-pass filter, as shown in Equation (4). Let *S*(*n*) represent the sound signal, *n* be the time coefficient, in this case α = 0.95:
  (4) $$\overset{\wedge }{S}(n)=S(n)-\alpha \cdot S(n-1)$$
(4) Hamming window: Discontinuity on both sides of the sound frame will produce additional signals, so the continuity of the audio spectrum will be destroyed. Window processing of the sound frames lowers the additional high-frequency signals on both sides of the sound frame to highlight the major signals at the center of the frame. By mixing, sound frame overlaps can produce the effect of continued border changes. Let *N* be all sampling points of a frame. A Hamming window is generally used to prevent overly dramatic changes in a window, as follows:
  (5) $$W(n)=\{\begin{array}{cc} 0.54-0.46\times \cos\left(\frac{2n\pi }{N-1}\right) & \text{, 0}\leq n\leq N-1\\ 0 & \text{,otherwise } \end{array}$$
(5) Fast-Fourier transform (FFT): FFT is the most commonly used sound signal processing technique. Let *k* be currently sampling point and *n* for all *N* sampling points within a period. It converts time domain signals into the frequency domain to facilitate energy distribution in the frequency spectrum, as shown below:
  (6) $$X[k]=\sum_{n=0}^{N-1}\tilde{x}[n]\exp(-j\frac{2\pi nk}{N}),\text{ 0}\leq k<\text{N}-1$$
(6) Triangular pass filter: The human auditory system can perceive frequencies ranging from 20–20,000 Hz, but is not equally sensitive to each frequency. The human ear is relatively sensitive to the low-frequency range, and less sensitive to changes at higher frequencies. In the sound identification system, the Mel-scale frequency is similar to the perceived frequency, and is the most commonly used simple frequency scale transformation equation, as described below.
  (7) $$Mel=2595\times \log\left(1+\frac{f}{700}\right)$$

**Figure 14:**
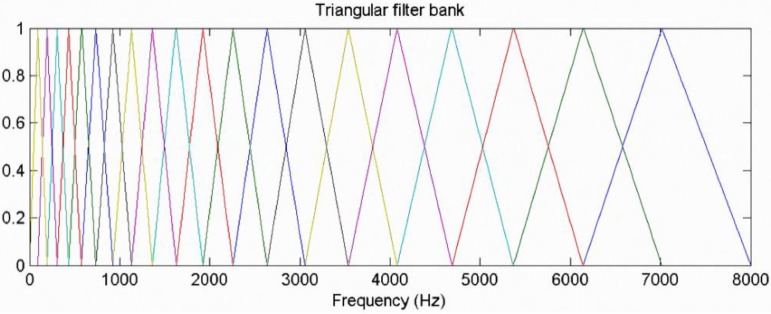
Triangular filter bank model.

Let *Mel* be the Mel frequency and f be the actual frequency. As shown in Figure 14, the triangular filter bank consists of a number of triangular band-pass filters. The design is based on the characteristics of the human ear. In the low-frequency part, the interval of the triangular filter bank is closer and the bandwidth also increases with frequency. The interval and frequency width will also increase accordingly, so it can simulate human ear characteristics, *i.e.*, it is more sensitive to lower frequencies. Using the Mel filter to process the signals of each frame, we obtain the signal frequency energy value, as shown below. 

Let *e*(*i*) be the *i* energy of No. *i* triangular filter, *l* be the number of triangular filters, φ be the function of No. Triangular filter *i*, and *A_k_* be frequency energy value of *S*[*k*]. As the signals after FFT are vertically asymmetrical, it is only necessary to compute in the range of *N*/2:

(8) $$e(i)=\sum_{k=0}^{\frac{N}{2}-1}\varphi_{i}(k)A_{k},\text{ 0}\leq i<l$$

(9) $$A_{k}={\left|S[k]\right|}^{2},\text{ 0}\leq k<\frac{N}{2}$$

(7) Discrete cosine transform (DCT): After obtaining the filter energy, we can calculate its logarithm value and enter it into the discrete cosine transform to get the M orders of characteristic coefficients. In this paper, M = 26. The DCT equation is shown in Equation (10).

Let *e*(*i*) be the energy value of No. *i* triangular band pass filter, *C_m_* be the *m*th order MFCC, L be the number of MFCCs (here, this = 12), and *E_k_* be the energy value after FFT computation:

(10) $$C_{m}=\sum_{k=1}^{M}E_{k}\cdot \cos[m\cdot (k-\frac{1}{2})\cdot \frac{\pi }{M}],\text{ m = 1, }...\text{, L}$$

(11) $$E_{k}=\log[e(i)]$$

(8) Delta cepstrum coefficients: MFCC does not produce accurate identification results. Thus, in addition to the 12th-order MFCC, we added the energy of the logarithm of the sound frame to get the 13th-order characteristic parameter, by obtaining the first-order differential cepstrum coefficients and the second-order differential cepstrum coefficients of the 13th characteristic parameters. We obtained a total of 39 orders of coefficients to represent the sound frame MFCC. The meaning of the differentiation is the change in the coefficients over time. The equation of differentiation is shown in Equation (12):
  (12) $$\Delta C_{m}(t)=\frac{\sum_{\tau =-M}^{M}\tau \cdot C_{m}(t+\tau )}{\sum_{\tau =-M}^{M}\tau^{2}},\text{ m = 1, }....\text{, L}$$

### 2.6. K-Means Algorithm 

Among the data-segmented clustering methods, the most widely used and commonly known method is K-means clustering, also known as ‘Forgy’s algorithm’ [23]. The main objective of K-means is to process a large number of high-dimension data to find representative data. These representative data are also known as cluster centers. These cluster centers can be used to carry out data classification and compress large amounts of data. When using K-means clustering, it is necessary to determine the number of clusters and gradually reduce the errors in the cluster after repeated itinerary computation until the errors do not change and converge to the final clustering results. The steps of implementing the K-means algorithm are as follows.

If the training sample is *x*^(*i*)^:

$$x^{\left(i\right)}=\{x^{\left(1\right)},x^{\left(2\right)},....,x^{\left(m\right)}\},\;x^{\left(i\right)}\in ℜ$$

(1) We randomly select K cluster centers as *μ_j_*:
  $$\mu_{j}=\{\mu_{1,}\mu_{2},....,\mu_{k}\},\;\mu_{j}\in ℜ$$
(2) Repeat the following process until convergence:
  (a) For each *x*^(*i*)^, compute the nearest cluster center and assign it to the nearest cluster center.
    (13) $$c^{\left(i\right)}:=\underset{j}{\text{argmin}}{\left\|x^{\left(i\right)}-{\text{μ}}_{j}\right\|}^{2}$$
  (b) For each category *μ_j_*, re-compute the mean value of the category and update the cluster center.
    (14) $$\mu_{j}=\frac{\sum_{i=1}^{m}\{c^{\left(i\right)}=j\}x^{\left(i\right)}}{\sum_{i=1}^{m}\left\{c^{\left(i\right)}=j\right\}}$$

**Figure 15:**
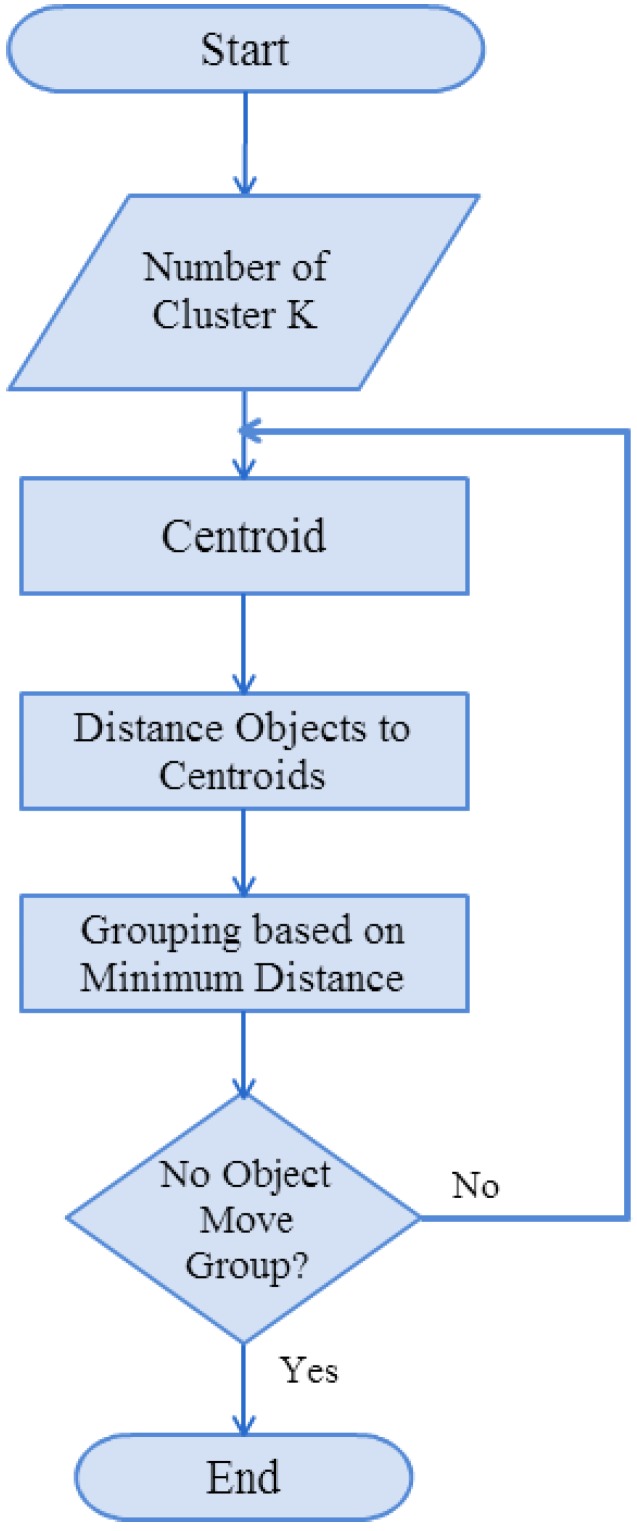
Flowchart of K-means algorithm.

Figure 15 shows the K-means algorithm process. It determines the cluster number K and establishes the cluster center according to the value of K before computing the distance of each data point from the cluster center, and assigns it to the nearest cluster center. After the distribution, a new cluster center is computed for distribution until the distance between the new cluster center and data satisfies the ending condition to complete the clustering process.

### 2.7. K-Nearest Neighbor Algorithm 

The K-nearest neighbor algorithm is based on the idea of ‘clustering things of the same nature’—in other words, objects of the same category should be closer in distance. The implementation process of the K-nearest neighbor algorithm is as follows:

(1) First, determine the number of nearest points of test data x against training data K using a Euclidean distance equation to compute the distance. If there are two points in k dimensional space, *x =* [*x_1_*, *x_2_*, *…*, *x_k_*] and *y =* [*y_1_*, *y_2_*, *…*, *y_k_*], the Euclidean distance between the two can be represented by Equation (15):
  (15) $$d(x,y)=\sqrt{\sum_{i=1}^{k}{\left(y_{i}-x_{i}\right)}^{2}}$$
(2) When test data x has more representatives than a certain category of data (the number of K-nearest points accounting for the majority), it is judged that x is of the certain category.

## 3. Experiments

### 3.1. Experimental Data 

We performed six experiments. The training data were sound files provided in [24]. Each training data sample included respiratory cycle data. Table 2 lists the experimental data format, consisting of 20 training data sets, training data times of 10–20 s, and MFCC of 39 dimensions. The test data for Experiments 1–4 used sound files from [24]. The test data in Experiment 5 were lung sound signals from subjects.

**Table 2:** Experimental Data.

KNN (K = 1) K-Means (K = 256)	Training Data	Test Data
Number	20	According to experimental conditions
Time (s)	10–20	10–20
MFCC dimensions	39	39
Category	Four categories (normal sounds, crackles, wheezes, and rhonchi)	No prior classification

### 3.2. Experimental Environment 

#### A. Experiment 1

Experiment 1 included two different conditions: (1) with the K-means algorithm; and (2) without it. We observed how two conditions affected the lung sound signal identification rate.

#### B. Experiment 2

Experiment 2 assumed that the experimental environment was ideal: we observed how this affected lung sound signal identification to determine whether the training data had converged.

#### C. Experiment 3

Experiment 3 assessed how environmental interference factors affected the identification of lung sound signals.

#### D. Experiment 4

Experiment 4 evaluated the health of lungs and computed the percentages of three kinds of abnormal lung sounds.

#### E. Experiment 5

We used the proposed system to record human lung sound signals to assess identification results of lung sound signals. Based on measurement data from a Bluetooth electronic stethoscope (Littmann 3200, 3M), we computed the error between data measured using the proposed system and the Littmann 3200 data. 

#### F. Experiment 6

Experiment 6 used the bending-type sensor to measure subjects’ respiratory states and computed the respiratory cycle (times/min) (the breathing rate of a normal adult is 12–20 times/min) to test the respiratory rate abnormal warning system.

### 3.3. Experimental Results

#### 3.3.1. Experiment 1 

**Figure 16:**
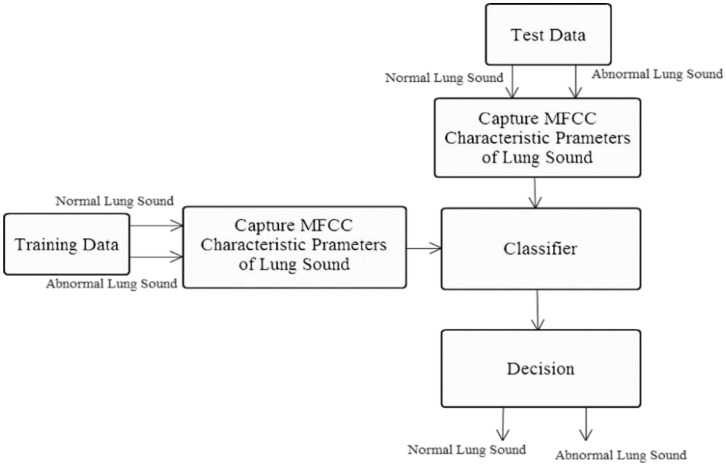
System A: Lung sound identification system.

This experiment assessed how combining MFCC and K-means algorithm affected the recognition of lung sound signals. The experiment was divided into two parts (A, B). System A (Figure 16) used the lung sound identification system without the K-means algorithm clustering. 

**Figure 17:**
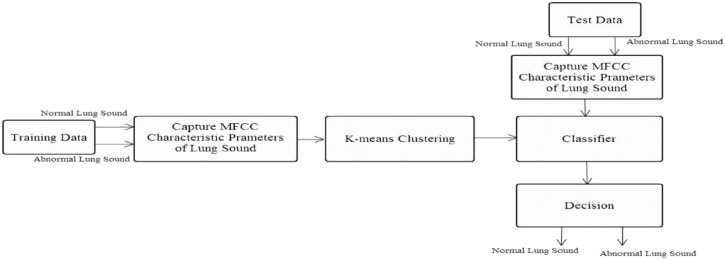
System B: Lung sound identification system with K-means algorithm clustering.

System B (Figure 17) used the lung sound identification system with the K-means algorithm clustering. To prevent other factors from affecting the identification results, identical sound files were used in the two training samples. Test sound files included 20 lung sound signals with 20 dB white Gaussian noise. The identification results of the two methods were compared.

**Table 3:** Identification results of Systems A and B.

Test Sound	System A (without K-Means) Identification Rates	System B (with K-Means) Identification Rates
20dB_AWGN normal lung sound (normal lung sound)	80.2%	95%
20dB_AWGN abnormal lung sound (crackles)	78.5%	92%
20dB_AWGN abnormal lung sound (wheezes)	79.6%	90.5%
20dB_AWGN abnormal lung sound (rhonchi)	70.3%	91.5%
Average identification rate	77.1%	92.3%

Note: All additional data used for current study including source code, normal lung sound, crackles, wheezes, rhonchi and the five clinical asthmatics audio archives are also shown in our project website [25].

As shown in Table 3, the addition of the K-means algorithm improved identification rates in System B by 15.1% compared with System A (without K-means). Table 4 compares training and testing times; without the K-means algorithm, despite the shorter training time, the testing time of System A was far longer than that of System B. Together, these experimental results demonstrated that combining MFCC and the K-means algorithm enabled good identification of lung sound signal, so System B was used for Experiments 2–6.

**Table 4:** Training and testing times of Systems A and B.

	System A (without K-Means)	System B (with K-Means)
Training time	0.17 s	98.2 s
Testing time	10.6 s	0.75 s

#### 3.3.2. Experiment 2 

This experiment evaluated the proposed system’s identification rates of lung sound signals in an ideal environment with no human interference. Test sound files (normal and abnormal) were used in the identification; Table 5 lists the results. KNN identification rates were 100%.

**Table 5:** Identification results of normal and abnormal lung.

	Sound File	Identification Rate
Normal lung sounds (Normal lung sounds)	Normal1.wav—Normal10.wav	100%
Abnormal lung sounds (crackles)	Crackles1.wav—Crackles10.wav	100%
Abnormal lung sounds (wheezes)	Wheezes1.wav—Wheezes10.wav	100%
Abnormal lung sounds (rhonchi)	Rhonchi 1.wav—Rhonchi 10.wav	100%

Note: All source archives of the normal lung sound, crackles, wheezes, rhonchi are for experimental measurements are shown in our project website [25].

#### 3.3.3. Experiment 3 

This experiment extended on Experiment 2. During stethoscope use, considerable noise may be added to lung sound signals due to changes in position, friction between clothing and the stethoscope, and touching the wire and tube. This experiment was designed to test whether the proposed system can eliminate this noise from lung sound signals to ensure results are correctly identified. We used lung sound signals and 20 dB white Gaussian noise as signal sources in the identification of lung sound signals. Table 6 lists the experimental results: for lung sound signals mixed with 20 dB white Gaussian noise, the normal lung sound average identification rate was 95%, while for abnormal lung sounds, it was 91.3%.

**Table 6:** 20 dB white Gaussian noise with normal and abnormal lung sounds.

	Sound File	Identification Rate
AWGN normal lung sound (normal lung sound)	AWGN_Normal1.wav—AWGN_Normal 10.wav	95%
AWGN abnormal lung sound (crackles)	AWGN_Crackles1.wav—AWGN_Crackles 10.wav	92%
AWGN abnormal lung sound (wheezes)	AWGN_Wheezes1.wav—AWGN_Wheezes 10.wav	90.5%
AWGN abnormal lung sound (rhonchi)	AWGN_Rhonchi 1.wav—AWGN_Rhonchi 10.wav	91.5%

Note1: All source archives of the normal lung sound, crackles, wheezes, rhonchi are for experimental measurements are shown in our project website [25].

#### 3.3.4. Experiment 4 

In this experiment, we classified lung health by grades according to the classification results of testing sound frames. Table 7 lists the grading descriptions. Sound frames are segmented into 10 segments, each of which is classified, and the classification results are analyzed in terms of the proportion of abnormal lung sound segments (an abnormal segment is labeled as 1, and a normal segment as 0). For example, if 0–2 of 10 sound frame segments are classified as abnormal, the sound frame is labeled as Good. If 3–5 segments are labeled as abnormal, the sound frame is classified as a Warning. If 6–8 segments are classified as abnormal, the sound frame is classified as Bad. If 9 or 10 segments are classified as abnormal, the sound frame is classified as Serious.

**Table 7:** Grading descriptions of lung sounds. (0 = normal lung sound; 1 = abnormal lung sound.)

Code Number	Degree	Condition
0000000000–0000000011	80%–100%	Good
0000000111–0000011111	50%–70%	Warning
0000111111–0011111111	20%–40%	Bad
0111111111–1111111111	0%–20%	Serious

**Figure 18:**
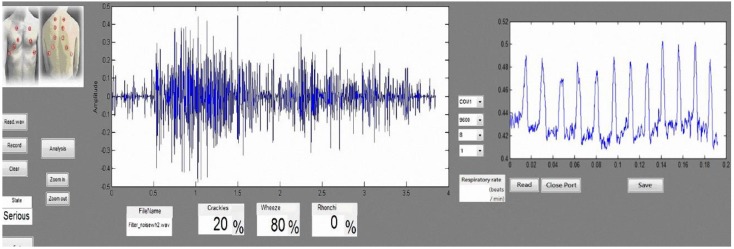
User interface for prevention and diagnosis.

For patients with composite problems, we used a mixer to mix the two types of abnormal lung sounds as the simulation signal: during the experiment, we selected a 10-s blank sound frame and mixed two types of abnormal lung sound (the first 2 s of crackles and the remaining 8 s of wheezes). The segment of abnormal lung sound after sound mixing was displayed on the user interface (Figure 18). Based on the experimental results, the system identified the results of the abnormal lung sound segment, with crackles accounting for 20% and wheeze for 80%, and the state being listed as Serious. In other words, the system can identify the segment of the abnormal lung sound accurately and process the abnormal signals of a patient with composite problems, as well as digitally displaying the results on the user interface to provide information about the state of the user’s lung health.

#### 3.3.5. Experiment 5 

**Figure 19:**
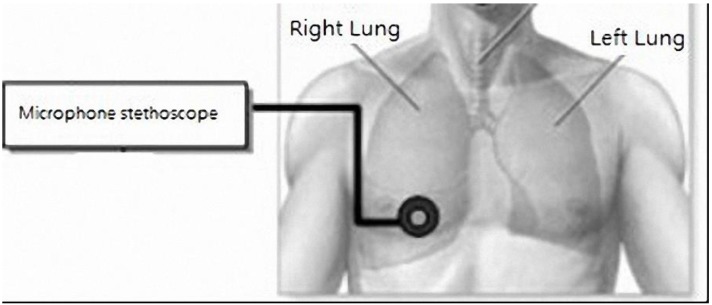
Lung auscultation position.

This experiment compared error rates between the results measured using the proposed stethoscope and those provided by a 3M Bluetooth electronic stethoscope (Littmann 3200) [26]. For lung auscultation, the proposed stethoscope was placed on the right section of the middle of the rib (Figure 19). To avoid any effects of gender or age, all subjects were males aged around 24 years old. They were all requested to go to bed early the night before the test, so all were fully rested. To help ensure the experimental data were objective, none of the subjects smoked or consumed any substances (e.g., tea, alcohol, pharmaceuticals) that might affect the test results.

**Figure 20:**
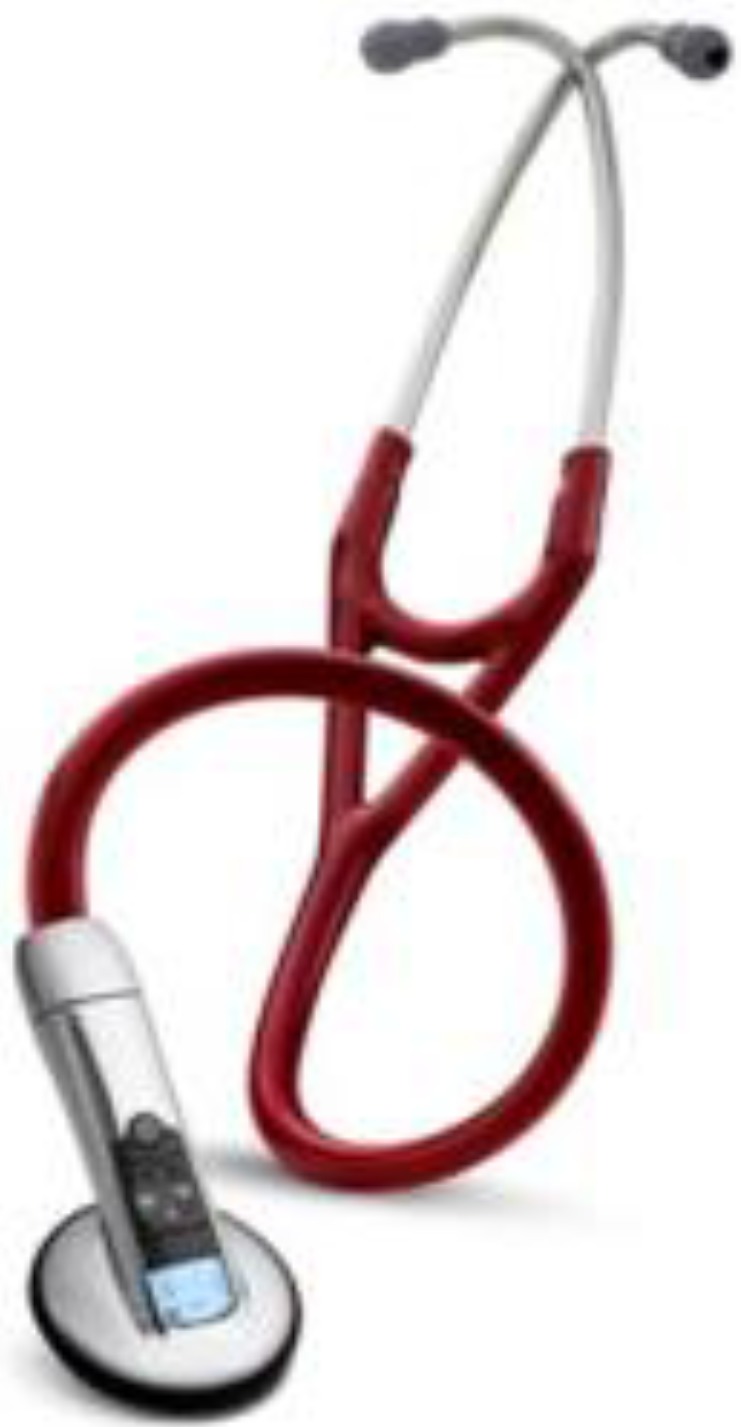
Littmann 3200 Bluetooth electronic stethoscope (3M) [26].

1. Comparison of device error: Table 8 lists the measurements and errors of the proposed stethoscope system, using a commercially available 3M Bluetooth digital stethoscope (Littmann 3200; see Figure 20) [26].

2. Verification method: In vector space, the most commonly used tool to judge the similarity of two vectors is the value of the cosine function of the angle between the vectors, as shown in Equation (16).

(16) $$\cos\text{θ}=\frac{X\cdot Y}{\left|X\right|\left|Y\right|}=\frac{\sum_{i=1}^{n}x_{i}\cdot y_{i}}{\sqrt{\sum_{i=1}^{n}{x_{i}}^{2}}\cdot \sqrt{\sum_{i=1}^{n}{y_{i}}^{2}}}$$

Let θ be the angle between two vectors of *x =* [*x_1_*, *x_2_*, *…*, *x_k_*] and *y =* [*y_1_*, *y_2_*, *…*, *y_k_*]. When the angle is smaller, the cosine function is closer to 1, indicating the two are more similar. The verification steps are as follows:

(1) Obtain lung sound waveforms from the 3M stethoscope and the modified microphone stethoscope.
(2) To combine the amplitudes of the waveform in various vertical lines to form the vector of the waveform signal, *X* = (0.0078, 0.00625, 0.054, ..., 0.23), *Y* = (0.0078, 0.0078, 0.0078, ..., 0.24). Let *X* vector be the 3M stethoscope and *Y* vector be the proposed stethoscope. *X* and *Y* formed the vector of the waveform.
(3) Enter the waveform vector into the cosine function and compute the degree of similarity of the two.

We used the two devices to record four groups of lung sound signals. Following the steps above, we computed the cosine function values (similarity) as shown in Table 8: 0.96, 0.97, 0.95, and 0.95, respectively. The average value was 0.956, *i.e.*, the degree of similarity between the output waveform of the two devices was 95.6%. Thus, the error between the proposed system and the 3M stethoscope is 4.4%. Table 9 compares the results from the two devices.

**Table 8:** Comparison of cosine function values between the two devices.

Vector	cosθ
<*X1*,*Y1*>	0.96
<*X2*,*Y2*>	0.97
<*X3*,*Y3*>	0.95
<*X4*,*Y4*>	0.95
Average	0.956

**Table 9:** Comparison of error between the two devices.

Product	Price	Sound Storage	Waveform Display	Simple Diagnosis	Error of Lung Sound Measurement Using The Devices (%)
3M Littmann 3200	20,000	Available	Computer display	Unavailable	4.4
Modified stethoscope	2000	Available	Computer display	Available	

We used the proposed system to measure the lung sounds of 20 subjects, and transmitted the amplified signals to the computer for analysis. Table 10 lists the basic data of subjects, and Table 11 lists the measurement results.

**Table 10:** Basic information of twenty subjects.

Subject	Gender	Age	Height (cm)	Weight (kg)	Coughing in the Last 7 Days	Family History of Disease	Measurement Posture
A	Male	24	170	64	none	No	Sitting
B	Male	24	180	107	none	Hypertension	Sitting
C	Male	24	171	70	none	No	Sitting
D	Male	24	163	55	none	No	Sitting
E	Male	25	172	90	none	No	Sitting
F	Male	25	181	69	none	Hypertension	Sitting
G	Male	23	177	67	none	No	Sitting
H	Male	23	172	75	none	No	Sitting
I	Male	24	172	54	none	No	Sitting
J	Male	24	172	52	none	No	Sitting
K	Male	24	163	61	none	No	Sitting
L	Male	24	177	64	none	No	Sitting
M	Male	24	172	71	none	No	Sitting
N	Male	24	172	68	none	No	Sitting
O	Male	25	173	68	none	No	Sitting
P	Male	25	176	69	none	No	Sitting
Q	Male	23	185	68	none	No	Sitting
R	Male	23	169	59	none	No	Sitting
S	Male	24	171	58	none	No	Sitting
T	Male	24	170	67	none	No	Sitting

**Table 11:** Analysis results of the current proposed system.

	Subject	Identification Results
Condenser microphone recordings	A	Good
	B	Good
	C	Good
	D	Good
	E	Good
	F	Good
	G	Good
	H	Good
	I	Good
	J	Good
	K	Good
	L	Good
	M	Good
	N	Good
	O	Good
	P	Good
	Q	Good
	R	Good
	S	Good
	T	Good

The current proposed system was used to measure the clinical asthmatics obtained from Cardinal Tien Hospital. There are five audio files, as shown in Table 12, Record1.wav, ..., Record5.wav, which are the auscultation recordings for different parts of the patient chest. The average recognition rate for asthma was 97.6% as shown in Table 12. 

**Table 12:** Asthmatics analysis results for the clinical asthmatics.

Audio file name	The average recognition rate
Record1.wav	97.6%
Record2.wav	
Record3.wav	
Record4.wav	
Record5.wav	

Note: The 5 source clinical asthmatics audio archives for experimental measurements are also shown in the website [25].

#### 3.3.6. Experiment 6 

##### Measurement Experiment with Wireless Respiratory Rate Detection System

**Figure 21:**
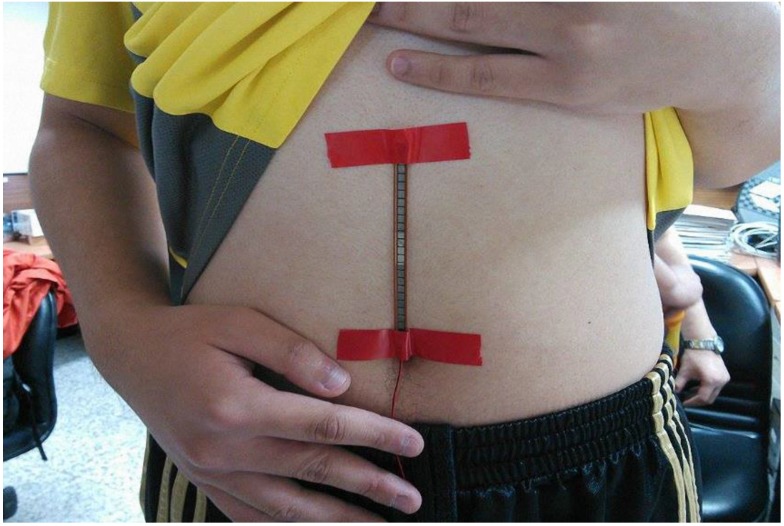
Position for the placement of the wireless respiratory rate detection system.

When using the wireless respiratory rate detection system, the bending-type sensor should be placed on the center of the abdomen (Figure 21) because changes in expansion and contraction are most apparent at this position.

##### Wireless Respiratory Rate Detection System Software

**Figure 22:**
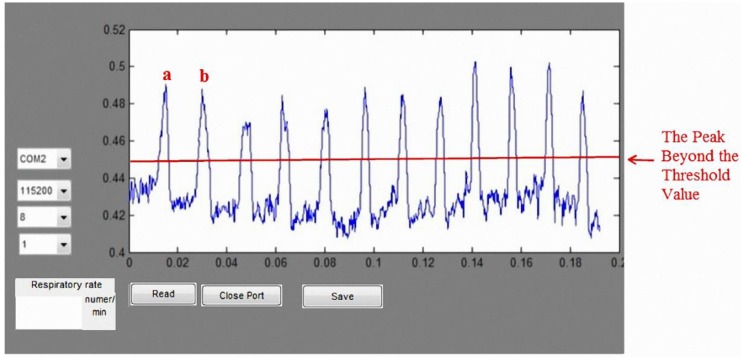
Wireless auscultation system user interface.

The GUI of the wireless respiratory rate detection software was developed using Matlab. After the hardware receives the respiratory signal, the Bluetooth wireless module sends the respiratory signals to the computer. Matlab reads the respiratory data from the virtual COM port. After regrouping the data, the data are stored and displayed for analysis. Figure 22 illustrates the user interface of the wireless respiratory rate detection system. After setting the threshold values, we can compute the number of peak values beyond the threshold value. As shown in Figure 22, “a” waves and “b” waves greater than the threshold values can be considered as the two respiratory cycles.

## 4. Discussion

### 4.1. Detection Accuracy 

We used the bending-type sensor to detect the breathing state and compute respiratory cycle (times/min) using the mean absolute percentage error (MAPE) as the evaluation indicator, as shown in Equation (17):

(17) $$\text{MAPE}=\frac{1}{M}\overset{M}{\underset{k=1}{\sum }}\left|\frac{x\left(k\right)-x^{\prime }(k)}{x(k)}\right|\times 100\%$$

Let (*k*) be the real value and *x*’(*k*) be the measured value. The principle of mean absolute percentage error (MAPE) can be applied here, as described previously [27] (see Table 13). The MAPE rate of the proposed respiratory detection system was 6.8%, indicating that the proposed respiratory detection function has high accuracy. Table 14 presents the results of the respiratory rate measurement, which demonstrate that the respiratory detection system can detect the breathing state of patients with asthma (respiratory rate > 25 times/min) [28] and send a warning about abnormal respiratory cycles.

**Table 13:** MAPE accuracy principles.

Model Prediction Capability	MAPE (%)
Highly accurate	10%
Good	10%–20%
Reasonable	20%–50%
Inaccurate	>50%

**Table 14:** Respiratory rate measurement results.

Subject	Sensing Respiratory Cycle (Times/Min)	Actual Respiratory Cycle (Times /Min)	MAPE Value
**A**	**17**	**15**	**6.8%**
B	16	15	
C	18	17	
D	15	16	
E	17	19	
F	14	14	
G	16	15	
H	16	17	
I	14	15	
J	15	14	
K	16	14	
L	17	17	
M	16	17	
N	15	14	
O	16	17	
P	14	16	
Q	16	17	
R	17	16	
S	16	15	
T	14	15	

### 4.2. Comparison with Existing Auscultation Systems 

Table 15 compares the results of the proposed system with (1) a traditional stethoscope (CK625P) [28] produced by Spirit; and (2) the 3M Littmann 3200 Bluetooth electronic stethoscope.

**Table 15:** Comparison with existing auscultation systems.

	Price (USD)	Sound Storage	Frequency Range	Waveform Display	Recording Time (Second )	Capturing Function	Simple Diagnosis	Wireless Function
CK625P [29]	60	Unavailable	20–10,000 Hz	Unavailable	Unavailable	Unavailable	Unavailable	Unavailable
3M-3200 [26]	667	Available	20–1000Hz	Computer display	10	Unavailable	Unavailable	Bluetooth transmission
Proposed system	73	Available	200–2000 Hz	Computer display	20	Available	Available	Unavailable

As shown in Table 15, the proposed system combines the advantages of a traditional analog stethoscope and a digital stethoscope. It can readily detect the respiratory rate and diagnose whether lung sound signals are abnormal. Thus, it has greater potential for application than the other digital auscultation systems.

## 5. Conclusions

In this study, we used mel-frequency cepstral coefficients (MFCCs) to capture lung sound signal characteristic parameters, along with the K-means algorithm and nearest-neighbor classification (Kth nearest neighbor), to establish a stethoscope system for detecting abnormal lung sounds (crackles, wheezes, and rhonchi). Based on the experimental results, MFCC combined with the K-means algorithm was successfully able to identify lung sounds. In an ideal noiseless environment, the proposed system’s training data identification rate can be as high as 100%. The average identification rate of lung sound signals mixed with 20 dB white Gaussian noise was 92.25%., which is an improvement of ~8.6% compared with the results reported in [19], and ~16% compared with the results reported in [17]. We used a condenser microphone to modify a stethoscope. The difference in error rates between the proposed system and the commercially available 3M Littmann 3200 Bluetooth electronic stethoscope [26] (Table 9) was 4.4%. Then, compared with existing auscultation systems, our proposed system has the advantages, recording time, capturing function, and simple diagnosis, as shown in the results of Table 15. Hence, our proposed system can be used for home diagnosis, because it provides lung sound signal sound frame identification results to grade lung sounds (Good, Warning, Bad, Serious). If lung sounds are classified as Warning (abnormal lung sounds accounting for 30% of signals), the system sends a warning message to the user to seek medical advice.

We also designed a bending-type sensor to detect the respiratory state of the subject. According to the benchmark of mean absolute percentage error (MAPE), as proposed in [27], the proposed respiratory detection function is highly accurate: the error was ~6.8%. Data are transmitted to the computer through Bluetooth, where the respiratory cycle (times/min) is computed for real-time detection. The average respiratory rate of an adult is 12–20 times/min [30]. Using the normal respiratory rate as a threshold, the system sends a warning to the user when it detects an abnormal respiratory cycle in an asthma patient. Together, these results confirm that the proposed lung sound abnormal diagnosis system and wireless respiratory detection system can help clinicians diagnose lung problems in patients.
